# HEalthcare Robotics’ ONtology (HERON): An Upper Ontology for Communication, Collaboration and Safety in Healthcare Robotics

**DOI:** 10.3390/healthcare13091031

**Published:** 2025-04-30

**Authors:** Penelope Ioannidou, Ioannis Vezakis, Maria Haritou, Rania Petropoulou, Stavros T. Miloulis, Ioannis Kouris, Konstantinos Bromis, George K. Matsopoulos, Dimitrios D. Koutsouris

**Affiliations:** 1 Biomedical Engineering Laboratory, School of Electrical & Computer Engineering, National Technical University of Athens, 9 Iroon Polytechniou St., 15780 Athens, Greece; pioannidou@biomed.ntua.gr (P.I.); ivezakis@biomed.ntua.gr (I.V.); rpetro@biomed.ntua.gr (R.P.); smiloulis@biomed.ntua.gr (S.T.M.); ikouris@biomed.ntua.gr (I.K.); konbromis@biomed.ntua.gr (K.B.); dkoutsou@biomed.ntua.gr (D.D.K.); 2 Biomedical Engineering Laboratory, Institute of Communication and Computer Systems, National Technical University of Athens, 9 Iroon Polytechniou St., 15780 Athens, Greece; mhari@biomed.ntua.gr

**Keywords:** healthcare robotics, upper ontology, human–robot interaction, communication ontologies, collaboration frameworks, ethical compliance, IoT in healthcare

## Abstract

**Background:** Healthcare robotics needs context-aware policy-compliant reasoning to achieve safe human–agent collaboration. The current ontologies fail to provide healthcare-relevant information and flexible semantic enforcement systems. **Methods:** HERON represents a modular upper ontology which enables healthcare robotic systems to communicate and collaborate while ensuring safety during operations. The system enables domain-specific instantiations through SPARQL queries and SHACL-based constraint validation to perform context-driven logic. The system models robotic task interactions through simulated eldercare and diagnostic and surgical support scenarios which follow ethical and regulatory standards. **Results:** The validation tests demonstrated HERON’s capacity to enable safe and explainable autonomous operations in changing environments. The semantic constraints enforced proper eligibility for roles and privacy conditions and policy override functionality during agent task execution. The HERON system demonstrated compatibility with healthcare IT systems and demonstrated adaptability to the GDPR and other policy frameworks. **Conclusions:** The semantically rich framework of HERON establishes an interoperable foundation for healthcare robotics. The system architecture maintains an open design which enables HL7/FHIR standard integration and robotic middleware compatibility. HERON demonstrates superior healthcare-specific capabilities through its evaluation against SUMO HL7 and MIMO. The future research will focus on optimizing HERON for low-resource clinical environments while extending its applications to remote care emergency triage and adaptive human–robot collaboration.

## 1. Introduction

Healthcare robotics has brought transformative changes to the medical field, addressing long-standing challenges such as workforce shortages, the increasing demand for personalized care, and the need for operational efficiency. By integrating advanced robotic systems, healthcare providers can enhance care delivery while mitigating critical resource constraints. Applications of robotics span a broad spectrum, from eldercare and rehabilitation to surgical precision, patient monitoring, and hospital logistics, making robotics indispensable in modern medicine [1,2,3,4,5].

Despite their potential, robotic systems face significant hurdles, including the need for seamless human–robot interaction (HRI), integration into healthcare infrastructure, and adherence to strict ethical and regulatory requirements. Achieving these capabilities necessitates robust frameworks capable of enabling robots to operate autonomously while collaborating with human stakeholders in dynamic environments. Ontologies, structured frameworks for organizing and interpreting knowledge, have emerged as fundamental tools for addressing these complexities [6].

This study introduces HERON (HEalthcare Robotics’ ONtology), an upper ontology that stands out as a comprehensive and pioneering framework specifically designed to address the intricate and dynamic requirements of healthcare robotics. Its modular architecture supports dynamic, multi-agent environments by structuring domains such as communication, collaboration, safety, and robot classification. By serving as a foundational framework, HERON facilitates seamless integration and adaptability in diverse healthcare scenarios, bridging the gap between theoretical advancements and real-world applications. The manuscript evaluates HERON against current ontologies, but its goal is to demonstrate a validated semantic framework rather than perform a review. The readiness of HERON for real-world robotic applications is demonstrated through scenario-based instantiations as well as SPARQL reasoning and SHACL-enforced constraint evaluation. As part of this research, the HERON ontology has been made publicly accessible on Zenodo https://doi.org/10.5281/zenodo.15116119, last accessed 31 March 2025 under an open-access license. Also, a GitHub repository maintains version control and documentation through the following link: https://github.com/ioannidou135/HERON-Ontology (accessed on 28 April 2025).

### 1.1. The Role of Robotics in Healthcare

Healthcare robotics has demonstrated its capacity to revolutionize various domains of medical care, offering precision, efficiency, and adaptability. Table 1 highlights examples of healthcare robotics applications, showcasing their impact across eldercare, surgery, rehabilitation, patient monitoring, and hospital logistics.

Eldercare robotics, such as Paro Robot and Care-O-Bot, focus on enhancing emotional well-being and health monitoring for aging populations. In surgical applications, systems like the da Vinci Surgical System enable minimally invasive procedures with unparalleled precision, reducing complications and recovery times [3,4]. Rehabilitation technologies, such as Lokomat and ReWalk, facilitate motor function recovery and mobility, while patient-monitoring robots like Robear and TUG improve the efficiency of care through real-time tracking of vital signs [9,10]. Logistics robots, including Aethon TUG and Moxi, streamline the transport of medical supplies and waste, significantly reducing staff workload [16,17]. However, the success of healthcare robotics relies on overcoming significant challenges. Effective HRI demands not only advanced technological frameworks but also the ability to anticipate, interpret, and respond to human behaviors in real time [5,9]. Additionally, interoperability within healthcare infrastructures remains a critical concern. Robots must integrate seamlessly with existing systems, respect privacy and security standards, and ensure efficient coordination in multi-agent settings [13].

### 1.2. Challenges in Using Ontologies

Ontologies are fundamental tools in robotics, providing structured frameworks that enable robots to categorize, interpret, and process complex information in a manner analogous to human cognition. In the context of healthcare robotics, HERON emerges as a novel modular ontology, specifically developed to address key domains like communication, collaboration, safety, and robot classification, setting a new standard in the field of healthcare robotics [18]. However, the implementation of such frameworks is not without challenges. Ontologies serve as the backbone of knowledge organization in robotics, enabling systems to process, categorize, and interpret complex information efficiently. In the context of healthcare robotics, HERON epitomizes an upper ontology that structures and harmonizes critical domains, including communication, collaboration, safety, and robot classification [18]. However, implementing such sophisticated frameworks is not without challenges.

One major obstacle is the complexity of ontological frameworks. While HERON’s modularity allows for adaptability, deploying it in real-world scenarios often requires extensive alignment with other standards, such as SUMO (Suggested Upper Merged Ontology) and HL7 (Health Level Seven) [18]. This alignment is essential for interoperability but imposes a significant cognitive and technical burden on healthcare institutions, particularly those with limited resources or expertise [19]. Furthermore, existing ontologies often fail to address the dynamic and unpredictable nature of healthcare environments. For instance, robotic systems in hospitals must adapt to fluctuating workloads, changing patient needs, and the integration of new medical technologies. Current frameworks are frequently too rigid to scale or adapt effectively, limiting their practical utility [20]. Addressing these inefficiencies is particularly challenging when frameworks are designed for narrow applications, making them unsuitable for diverse healthcare scenarios. Another critical challenge lies in the limited versatility of existing ontologies. Robotic systems that excel in specific domains, such as eldercare, often struggle to adapt to other contexts, such as surgical robotics or hospital logistics. This lack of adaptability increases costs for healthcare providers, who may need to invest in multiple specialized robots rather than a unified system [8]. Lastly, the gap between theoretical research and practical implementation remains a persistent issue in healthcare robotics. While academic advancements focus on developing sophisticated frameworks, their deployment in real-world scenarios often faces significant challenges. HERON’s modular approach attempts to bridge this divide by streamlining communication and collaboration modules, enabling robots to integrate seamlessly into existing healthcare infrastructures [21]. This adaptability not only facilitates real-world deployment but also enhances interoperability with established standards such as IEEE or OWL/XML, fostering a cohesive and efficient healthcare ecosystem [22].

### 1.3. Objectives and Contributions

The primary objective of this research is to develop an upper-level HERON ontology that addresses critical challenges in healthcare robotics, including complexity, adaptability, and practical usability. By refining the communication and collaboration modules, the study simplifies the cognitive and technical requirements associated with deploying ontological frameworks. This approach ensures that HERON is accessible to a diverse range of stakeholders, such as clinicians, caregivers, and system integrators, promoting its broader adoption in real-world healthcare settings. The contributions of this study are closely tied to practical healthcare applications and can be summarized as follows:

1. **Simplifying Complexity**: Existing ontological frameworks often suffer from excessive complexity, which limits their deployment in healthcare settings. HERON addresses this challenge by streamlining its communication and collaboration modules, removing redundant features, and simplifying its design. For example, in eldercare scenarios, this streamlined design allows robots to perform tasks such as emotional monitoring or medication reminders without overwhelming caregivers with technical overhead. Similarly, the simplified modules enable surgical robots to integrate seamlessly into operating rooms, reducing the cognitive burden on surgeons and ensuring precision during complex procedures.
2. **Improving Adaptability**: Healthcare environments are dynamic and often unpredictable, requiring systems that can adapt to evolving patient needs, fluctuating workloads, and the integration of new technologies. HERON enhances adaptability by enabling robots to respond to nuanced cues, such as emotional signals in eldercare settings, and by supporting real-time data exchange in surgical contexts. For instance, robots equipped with HERON can prioritize tasks dynamically in emergency departments or adapt their workflows based on patient vitals in operating rooms, making HERON a versatile framework capable of addressing a wide range of healthcare scenarios.
3. **Bridging Academia and Industry**: Academic research on healthcare robotics tends to focus on theoretical advancements, while practical deployment remains underexplored. This study bridges this divide by providing a scalable, user-friendly ontology that transitions seamlessly from research prototypes to real-world applications. By facilitating the integration of HERON into healthcare settings, such as hospital logistics and diagnostic workflows, the framework promotes collaboration between academic research and industry practices. This ensures that HERON remains relevant in addressing both theoretical advancements and practical demands in healthcare robotics.
4. **Enabling Interoperability**: Effective integration of robotic systems into healthcare infrastructure requires interoperability with existing standards and technologies. HERON is designed to function cohesively within diverse healthcare ecosystems, adhering to recognized technology standards such as IEEE and OWL/XML. For example, HERON’s compliance with these standards allows eldercare robots to interact with electronic health records (EHRs) and IoT devices, ensuring synchronized data exchange. In multi-agent surgical teams, HERON facilitates seamless task distribution and collaboration between robotic and human agents, improving overall procedural outcomes.

The upper-level HERON ontology proposed in this research addresses long-standing challenges in healthcare robotics by reducing complexity, improving adaptability, bridging theoretical and practical gaps, and enabling interoperability. By focusing on real-world applications such as eldercare and surgical robotics, HERON establishes itself as a transformative framework, paving the way for more cohesive and efficient robotic systems in diverse healthcare contexts.

While the manuscript surveys related ontologies for comparison, its core contribution lies in presenting HERON as a modular, validated framework designed through instantiations and SHACL-enforced reasoning, thereby bridging theoretical modeling with practice.

## 2. Background

### 2.1. Ontologies and Their Role in Robotics

**Figure 1:**
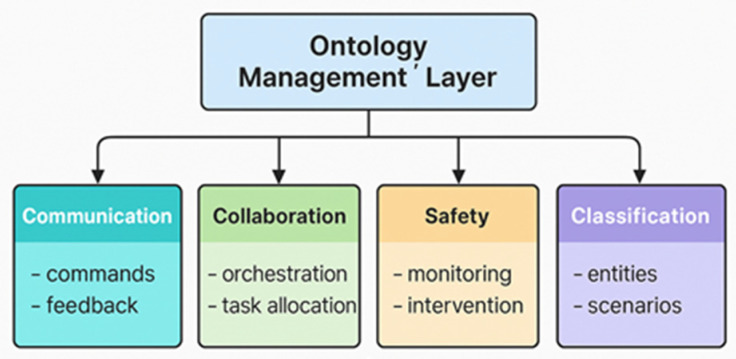
Modular architecture of the HERON ontology, showing its four semantic domains (communication, collaboration, safety, and robot classification), managed through a central ontology management layer.

Ontologies form the backbone of knowledge representation in robotics, enabling systems to process, categorize, and interpret information in a structured and meaningful way. In healthcare, where precision, interoperability, and adaptability are critical, ontologies such as HERON, SUMO, and HL7 provide frameworks that bridge gaps between disparate systems. HERON, uniquely positioned as an upper ontology for healthcare robotics, pioneers the integration of communication, collaboration, safety, and robot classification domains into a unified, modular structure. This modularity makes HERON flexible and scalable, allowing it to adapt to various healthcare scenarios while aligning with the goals of improved efficiency, enhanced patient outcomes, and increased stakeholder trust [23,24]. Figure 1 illustrates HERON’s modular architecture, emphasizing its core components: communication, collaboration, safety, and robot classification. These interconnected modules support seamless task management in healthcare robotics.

HERON stands apart from general-purpose frameworks such as SUMO (Suggested Upper Merged Ontology), which provides a broad foundational framework for interoperability across domains. While SUMO ensures robust communication between robotic systems and broader technologies, it lacks the domain-specific focus required for healthcare applications. In contrast, HERON’s tailored design meets the nuanced demands of healthcare robotics, enabling precise task allocation and real-time collaboration [25,26]. Similarly, HL7 (Health Level Seven) excels in standardizing healthcare data for efficient clinical information exchange, yet its scope is largely confined to static data interoperability. HERON complements HL7 by introducing dynamic, context-aware capabilities that allow multi-agent robotic systems to perform tasks like patient monitoring or surgical assistance, thus addressing critical gaps in robotic interaction frameworks [24,27].

Despite these strengths, the implementation of ontologies often requires specialized technical expertise, which can hinder their widespread adoption. Furthermore, the dynamic nature of healthcare environments—marked by fluctuating workloads, evolving patient needs, and rapid technological advancements—tests the adaptability of existing frameworks. HERON mitigates these challenges through its structured yet flexible system, enabling real-time decision making and adaptation to changing scenarios. For instance, HERON-equipped robots can prioritize tasks dynamically based on patient vitals or surgical parameters, providing essential support to healthcare teams in high-pressure environments [27,28].

Table 2 highlights the strengths and weaknesses of prominent ontology frameworks. HERON’s modular design uniquely addresses healthcare-specific challenges, while SUMO and HL7 complement HERON by facilitating interoperability and standardization.

Ontologies are not merely tools for organizing data; they are essential for robotic autonomy and collaboration. Autonomy in healthcare robotics depends on a robot’s ability to make decisions based on real-time data while determining how to use the data effectively. Collaboration, particularly in multi-agent systems, is another critical area where ontologies like HERON excel. By standardizing information exchange, HERON enables coordinated actions among multiple robots or human–robot teams, allowing robots to conform to human workflows and improve overall efficiency [28].

### 2.2. Communication in HRI

Human–robot interaction (HRI) in healthcare hinges on clear and trusted communication. Communication ontologies, such as those integrated into HERON, allow robots to interpret and respond to human intentions and behaviors in contextually appropriate ways. HERON’s communication module structures these interactions using semantic rules and context-aware algorithms, enabling robots to tailor responses based on the emotional and cognitive states of elderly patients. For example, robots using HERON have provided companionship and emotional support in eldercare, fostering meaningful interactions that go beyond functional assistance [32,33]. In surgical applications, communication ontologies enable real-time synchronization between surgeons and robots. HERON-equipped robots can respond dynamically to verbal commands, gestures, and physiological cues, reducing the cognitive burden on surgeons. This capability allows surgeons to focus on complex decision making while robots handle repetitive or precision-based tasks. Additionally, communication ontologies facilitate multi-modal interaction, combining speech, visual, and environmental cues to ensure seamless collaboration [34,35].

Communication ontologies also improve patient–robot interactions by enabling meaningful dialogues. HERON has been deployed in domains where robots remind patients to take medications or guide them during physical therapy exercises. These interactions enhance not only the functional aspects of care but also the emotional well-being of patients, particularly elderly individuals experiencing social isolation. Furthermore, remote-controlled service robots have shown promising results in providing assistance and emotional support to elderly and disabled individuals, supporting continuous care, especially in partially supervised or home-based settings [7]. In surgical settings, HERON’s communication capabilities enable robots to provide real-time feedback to surgeons, such as identifying anomalies or suggesting alternative approaches [32,36].

Despite these advancements, significant challenges remain. The variability in human communication styles, shaped by linguistic, cultural, and individual differences, complicates the standardization of robot responses. Additionally, resource-constrained environments pose challenges in calculating convergence rates and integrating communication ontologies with existing healthcare technologies. Addressing these challenges requires continuous refinement of communication ontologies, emphasizing adaptability and user-centered design [33,35].

### 2.3. Collaboration in Multi-Agent Systems

Multi-agent systems (MASs) are pervasive in healthcare robotics, where seamless collaboration between multiple agents—both robotic and human—is essential. MASs must operate in dynamic environments with rapidly changing demands, driven by patient needs and institutional priorities. HERON, with its modular design, directly addresses the inherent challenges of MASs, including task allocation, information exchange, and conflict resolution [29]. This capability makes HERON especially suitable for managing the complexities of healthcare settings. A major challenge in MASs is synchronizing agents with diverse objectives and capabilities. For instance, an optimal surgical team often involves multiple robots complementing human surgeons, each performing precise, coordinated tasks. HERON addresses this through role-based task distribution, assigning responsibilities based on agent capabilities and overall procedural goals. This approach not only increases operational efficiency but also minimizes errors by assigning tasks requiring high precision or speed to robots while reserving complex decision making for human agents [37].

When compared to other frameworks, HERON demonstrates significant advantages. Unlike SUMO, which provides a broad high-level framework for ontology integration, HERON’s granular modules are tailored to address the specific demands of healthcare robotics [37]. Similarly, while OBO (Open Biomedical Ontologies) excels in biomedical data standardization, it lacks the comprehensive task management capabilities required for MASs in dynamic healthcare environments [31]. HERON’s specificity enables it to address real-world challenges, such as managing multiple agents in an emergency ward, where decisions must be made collaboratively and under significant time constraints. However, HERON’s modular design presents certain limitations. Its computational complexity requires substantial processing power, which may not be feasible in resource-limited environments [38]. Additionally, ensuring compliance with interoperability standards remains a challenge due to the proprietary nature of many robotic platforms [29]. Future work should focus on developing lightweight, adaptable versions of HERON that can integrate seamlessly into mature MAS infrastructures while reducing computational demands.

### 2.4. Ethical and Privacy Concerns

The integration of robotics into healthcare necessitates a focus on ethical and privacy considerations, given the critical need to protect patient safety and safeguard sensitive data. HERON breaks new ground by embedding ethical compliance and data protection as fundamental pillars of its modular architecture, setting it apart in its ability to address stakeholder concerns with unparalleled trust and accountability [39].

HERON’s approach to privacy centers on its alignment with stringent regulatory frameworks such as the General Data Protection Regulation (GDPR). This ensures that sensitive patient data processed by healthcare robots—such as monitoring and diagnostic systems—are securely managed and accessible only to authorized personnel. Through the implementation of role-based access controls (RBACs), HERON limits data access to agents based on their specific tasks, thereby preserving confidentiality in multi-agent healthcare environments [40]. A unique ethical challenge in healthcare robotics lies in delegating critical tasks to autonomous systems. HERON tackles this by incorporating real-time monitoring and fail-safe mechanisms within its safety module. These features allow robots to perform high-stakes operations, such as medication administration or surgical assistance, while maintaining an additional layer of oversight. Detailed operational logs ensure that all robotic actions are tracked and accessible to clinicians, enabling real-time intervention when autonomous decision-making falls short [41]. Unlike frameworks such as SUMO and HL7, which prioritize data standardization and interoperability, HERON integrates ethical safeguards and privacy measures as fundamental design principles. By embedding encrypted communication protocols, stringent access controls, and transparent operational logging, HERON sets a benchmark for ethical compliance in healthcare robotics [31]. Despite its strengths, HERON faces challenges in navigating diverse regulatory environments. Its strong alignment with the GDPR, while critical for privacy, may hinder its adaptability in regions with differing or less stringent data protection laws. Additionally, the rapid evolution of healthcare technologies continually introduces novel ethical considerations, requiring ongoing enhancements to HERON’s framework [39,42].

HERON is designed to complement healthcare interoperability standards, such as HL7 RIM and FHIR, by offering an ontological abstraction layer focused on task semantics, role policies, and collaborative behaviors. Its alignment with robotic middleware and hospital workflow logic allows seamless integration with EHR-centric infrastructures.

To address these challenges, a collaborative effort between roboticists, policymakers, and healthcare professionals is essential. By fostering interdisciplinary partnerships, HERON can evolve to maintain its relevance and efficacy in addressing emerging ethical and privacy concerns. This proactive approach positions HERON as a sustainable and ethical solution for the future of healthcare robotics.

## 3. Materials and Methods

### 3.1. HERON Ontology Design

To address the complex and diverse requirements of healthcare robotics, the HERON ontology was designed to be modular, scalable, and compatible with IEEE standards. Its architecture comprises four interconnected dimensions: communication, collaboration, safety, and robot classification. These components cater to specific domains of healthcare robotics, including interactions between robots, robots and humans, and robots with their environment. By adopting a modular design, HERON achieves the dual benefits of adaptability and targeted refinement, enabling seamless scalability across a wide range of robotic tasks—from eldercare to surgical applications [43].

#### 3.1.1. Modular Structure and Its Components

The modular design of HERON facilitates incremental advancements while maintaining system integrity, marking a significant departure from conventional frameworks. Each of the four dimensions serves a distinct purpose within the healthcare robotics ecosystem:

1. **Communication**: Facilitates seamless information exchange, both between robots and between robots and humans. This component ensures that robots can interpret and respond to commands, queries, and environmental stimuli effectively, enabling synchronized operations in multi-agent settings.
2. **Collaboration**: Focuses on task-sharing and cooperative behavior, whether between robots or between robots and human teams. This is critical in scenarios such as surgical operations, where precise coordination is required among multiple agents.
3. **Safety**: Encompasses protocols and measures to ensure the physical and operational safety of both patients and healthcare providers. It includes fail-safes and real-time monitoring to mitigate risks in dynamic environments.
4. **Robot Classification**: Categorizes robots based on their characteristics, capabilities, and functionalities. This dimension enables efficient deployment by matching specific robot types to appropriate healthcare tasks, enhancing operational efficiency.

**Figure 2:**
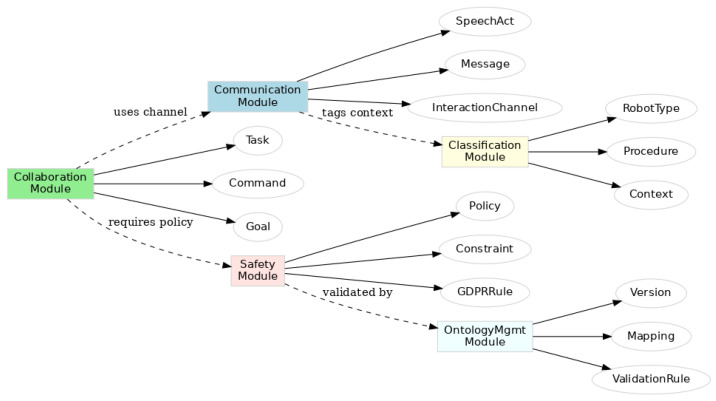
Graphical overview of HERON ontology modules and key semantic domains.

Figure 2 provides an overview of HERON’s modular ontology architecture.

The compartmentalization of these domains ensures that updates or refinements to one module can be implemented without affecting the others. This targeted approach allows HERON to evolve with the rapidly changing demands of healthcare robotics while maintaining its integrity [44]. The modular design of the ontology allows it to support various robotic applications including surgical guidance and rehabilitation feedback through scenario-based instantiations instead of a single universal ontology. The HERON framework avoids universal model imposition by enabling domain-specific instantiations which adapt the ontology to meet application-specific requirements in surgery, rehabilitation, and logistics.

#### 3.1.2. Compliance with IEEE Standards

HERON is developed in strict accordance with IEEE standards, ensuring compatibility with existing healthcare infrastructures. These standards are critical for establishing interoperability between HERON and other IEEE-compliant devices and systems. For example, HERON leverages IEEE guidelines for autonomous systems to enable standardized communication protocols and data exchange in multi-agent scenarios. This alignment is particularly vital in healthcare environments where seamless integration and interoperability are prerequisites for effective operation [44].

#### 3.1.3. Implementation Using OWL/XML

HERON is implemented using OWL/XML, a popular language for ontology representation that defines relationships and properties with fine-grained precision. Unlike HTML, the XML format offers compatibility across a wide range of applications and software platforms, making HERON both tractable and flexible. This implementation approach provides two distinct advantages:

1. **Integration Simplification**: OWL/XML simplifies the process of integrating HERON into diverse healthcare systems by offering a transparent structure that stakeholders can easily interpret.
2. **Enhanced Adaptability**: OWL/XML supports dynamic updates and real-time processing, enabling HERON to incorporate new data streams—such as patient vitals or imaging results—without requiring reprogramming. This adaptability is critical in healthcare environments, where conditions and requirements can change rapidly [45].

The HERON ontology, developed using OWL/XML, is publicly accessible on Zenodo (https://doi.org/10.5281/zenodo.15116119, last accessed 31 March 2025). Additionally, OWL/XML serialization ensures cross-platform interoperability, enabling HERON to function seamlessly within distributed technological ecosystems that include cloud computing systems and IoT devices. Generally, The OWL/XML format not only ensures compatibility with diverse platforms but also enables dynamic updates to accommodate evolving healthcare needs. The structure of the ontology also supports semantic mappings to existing EHR systems and robotic middleware platforms, which enables integration with hospital workflows and data pipelines.

The ontology was developed using Protégé, an open-source OWL ontology editor, which supports modular class construction, consistency checking, and SHACL shape creation. It was compatible with OWL-DL reasoners, which made it ideal for healthcare-specific ontological modeling.

#### 3.1.4. Future Directions in Design

Despite its many advantages, the implementation of HERON presents certain challenges. OWL/XML’s complexity can result in increased computational overhead, making deployment in resource-constrained environments difficult. Moreover, maintaining compliance with evolving IEEE standards and integrating HERON into proprietary robotic platforms remains an ongoing challenge. Addressing these limitations will require continuous optimization of HERON’s architecture to ensure its efficiency and effectiveness in diverse healthcare settings [45].

### 3.2. Validation and Testing

The HERON ontology was validated and tested through an instantiation-based approach, ensuring that its theoretical design could align with practical healthcare robotics applications. Rather than direct deployment in real-world systems, this method utilized simulations and scenarios derived from the Horizon 2020 ENDORSE project, demonstrating HERON’s ability to address safety, communication, collaboration, and task management challenges in complex healthcare environments [46]. By modeling specific use cases, the instantiations validated HERON’s adaptability and functionality across various healthcare operations.

#### 3.2.1. Instantiation Methodology

The validation approach was designed to ensure HERON’s modules were tested under controlled yet realistic conditions. This methodology focused on demonstrating how HERON could effectively address healthcare robotics challenges by simulating operational workflows, safety concerns, and communication protocols. Key steps included:

1. **Use Case Alignment:** The instantiations were closely aligned with healthcare scenarios to model real-world challenges. Examples include the following [47]:
  - Logistics Robots: Robots performed material transportation tasks in healthcare settings such as Fundació Ave Maria Healthcare Center, navigating shared spaces while interacting with human personnel.
  - E-Diagnostic Robots: Robots equipped with diagnostic modules supported patient monitoring and automated data collection. These simulations addressed critical aspects of robot–human collaboration in sensitive medical environments. By focusing on these scenarios, HERON’s modules were tested for scalability, adaptability, and safety in diverse operational contexts.
2. **Meta-Model Instantiation:** HERON’s design and functionality were validated against a meta-model structure, focusing on safety and operational components. For instance:
  - Robots like RB1-Base were instantiated as collaboration and interacting agent objects, reflecting their collaborative roles in shared tasks [47].
  - Safety-critical elements such as fault management (e.g., motion safety) were modeled using ISO-compliant standards for dependability and security [48].

Metrics such as mean time between failures (MTBFs) and mean time to recovery (MTTR) were included to validate HERON’s fault tolerance and system reliability under varying operational conditions.

3. **Stakeholder Integration:** Stakeholder recommendations were incorporated into the validation process to ensure HERON’s modules met both practical and theoretical requirements. This step emphasized safety protocols and integration strategies for multi-robot collaboration in healthcare facilities, ensuring the design was aligned with real-world applications [10,48].
4. **Key Performance Indicator (KPI) Evaluation:** HERON’s functional adequacy was evaluated through KPIs tailored to healthcare robotics. The metrics included the following:
  - The number of successful missions completed.
  - Distance traveled by robots during logistics tasks.
  - Error rates during navigation and task execution.
  - Energy efficiency, such as missions completed before requiring recharging.

These KPIs were used to measure HERON’s operational reliability, adaptability, and alignment with safety requirements in simulated healthcare workflows [47].

#### 3.2.2. Examples of Instantiation

The instantiations demonstrate use cases from the ENDORSE project, which include high-risk medication delivery, ethically constrained visitation, and medical coordination support. Each was simulated with HERON enforcing role-based logic and SHACL constraints. The use cases are the following:

1. **Logistics Use Case:** The logistics robots in Fundació Ave Maria Healthcare Center transported items such as linens and pharmaceuticals across multiple stations, operating in spaces shared with humans. HERON’s collaboration module ensured role-based task distribution, while its communication module facilitated seamless interactions between robots and their environment. Safety features, like fault management systems, mitigated risks such as erratic movement and disorientation during navigation [47].
2. **E-Diagnostic Use Case:** Robots equipped with e-diagnostic modules provided real-time patient monitoring and data collection. By leveraging HERON’s communication and safety modules, these robots could securely transmit medical data to electronic health records (EHRs), adhering to GDPR compliance through role-based access controls and encrypted communication protocols [47].
3. **Fleet Management System (FMS):** The FMS use case, as described in conference proceedings [48], modeled the collaboration of three RB1-Base robots tasked with material transport in a healthcare facility. HERON’s safety slice included tolerance levels for system monitoring and fault prevention mechanisms, such as routing analytics and motion safety algorithms. The instantiations validated HERON’s ability to manage multi-robot interactions while ensuring safety and efficiency under diverse operational conditions.

**Figure 3:**
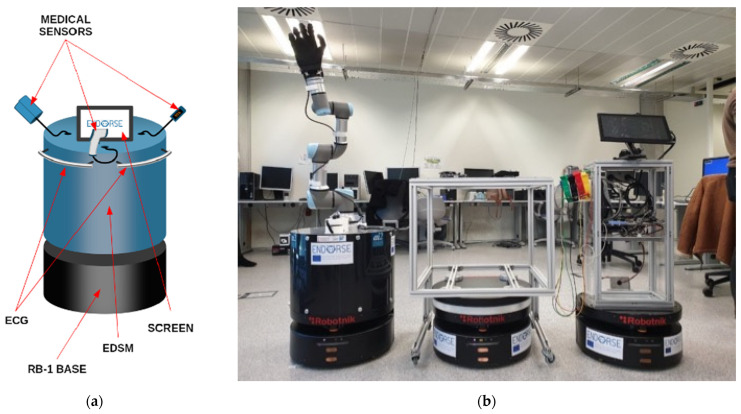
The ENDORSE robots. (**a**) Schematic representation of an ENDORSE robotic platform highlighting its modular components, including the RB-1 base, medical sensors (ECG, EDSM), and integrated user interface (screen); (**b**) Actual deployment of ENDORSE robotic platforms demonstrating their flexible configurations and capabilities for safe, collaborative tasks within healthcare research settings.

The HERON system requires realistic healthcare workflow implementation through simulated scenarios which include representative robotic agents. The system includes mobile visitation units and medication delivery robots and hospital logistics platforms. The ENDORSE project demonstrates HERON-enabled agents through the examples shown in Figure 3 when deployed for specific tasks.

Multiple agents with defined roles such as *MedicationAssistant*, *SupervisorBot*, and *VisitationDrone* were instantiated to exemplify the use of HERON in simulated scenarios. Each agent operated within the semantic constraints defined by the ontology’s modules. A *VisitationDrone* was allowed to enter a patient’s room only if the patient was awake and no restricted flags were active. Permission checking was performed using SPARQL queries over the instantiated RDF graph. SHACL validation shapes were applied in parallel to verify compliance with safety and institutional policies such as requiring human escort for specific risk profiles. OWL-based reasoning and SHACL constraints were used in a hybrid manner to enable agents to adapt their behavior dynamically according to situational context and institutional norms.

#### 3.2.3. Semantic Constraint Implementation

The real-time reasoning tasks of HERON became operational through semantic validation, which employed SPARQL queries and SHACL shapes. The mechanisms allowed agents to receive dynamic assessments of their behavior against role and context and policy constraints while the scenarios played out.

**Figure 4:**
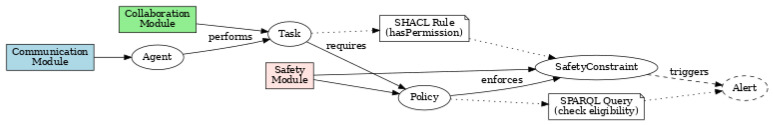
Semantic flow in the HERON ontology across modules. The diagram shows execution paths, policy enforcement, and validation using SHACL rules and SPARQL queries in real-time interaction scenarios.

Figure 4 shows the semantic interactions and validation flow across HERON modules using SHACL and SPARQL mechanisms.

**Figure 5:**
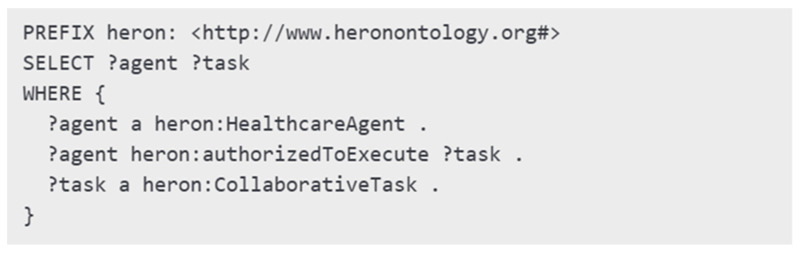
Example query SPARQL.

The SPARQL query shown in Figure 5 demonstrates how to check robotic agents for eligibility to perform medication delivery tasks. The query determines which healthcare agents have permission to execute particular collaborative tasks, which allows robots to perform actions through pre-validated institutional knowledge.

The *MedicationAssistant* robot needed both the *hasAuthorityToOverride* flag and an unconscious patient to activate its override capability for default delivery protocols. The SHACL model enforced this restriction to stop unauthorized overrides from happening. The *VisitationDrone* needed a human nurse escort for ICU room entry according to the *requiresHumanEscort* property. The ethical enforcement mechanisms of HERON operate both during design time and real-time task execution, as demonstrated by these examples.

**Figure 6:**
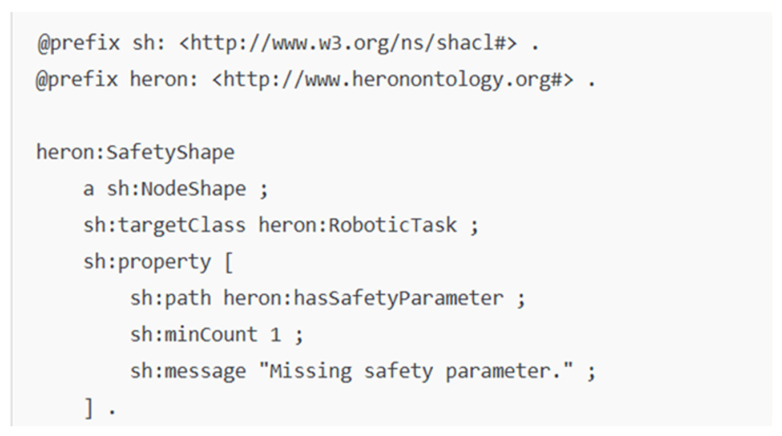
SHACL shape example.

The system uses SHACL shapes to enforce semantic constraints while validating actions. The shapes specify the required structural elements along with properties needed to execute policies correctly. A SHACL shape example presented in Figure 6 demonstrates the verification process for the agent role and authorization possession to meet institutional and ethical requirements.

During validation scenarios, HERON used SPARQL queries to perform dynamic reasoning about whether a *VisitationDrone* could reach a patient’s room when conditions such as *patientAwake* and *noRestrictedFlag* were present. The SHACL shapes validated structural constraints on agent roles and permissions. The robot needed to have the *hasConsentToShare* property and to meet its associated authorization shape to execute data transfer tasks while following GDPR regulations. The semantic rules allowed agents to execute contextually eligible actions in real time.

The consistency and satisfiability checks were performed using HermiT and Pellet reasoners in Protégé to ensure the logical soundness of class axioms and contextual policies.

#### 3.2.4. Metrics for Evaluation

The evaluation metrics summarized in Table 3 provided a structured framework for assessing HERON’s validation outcomes.

Τhe evaluation of HERON was based on semantic correctness and policy conformance rather than on numerical performance metrics. For each modeled scenario, SHACL constraints were used to check that the instantiated agents met the safety and ethical rules defined by the ontology. These included requirements such as having the appropriate role-based permissions to execute a task, meeting contextual conditions (e.g., patientAwake), or delegating actions in accordance with institutional protocols. SPARQL queries were used to test the system’s ability to infer valid task paths and whether preconditions were met before allowing agents to act. The results showed that HERON provides consistent and reliable semantic control, which supports dynamic decision making in healthcare environments with high regulatory and ethical demands.

#### 3.2.5. Future Directions in Validation

Validation through instantiations confirmed HERON’s suitability for current healthcare scenarios, showcasing its ability to address safety-critical challenges and operational constraints. By focusing on adaptability, scalability, and regulatory compliance, HERON demonstrated its potential as a foundational framework for healthcare robotics.

However, challenges such as computational overhead in resource-constrained environments and achieving universal scalability across heterogeneous systems remain areas for further optimization. Future work will aim to refine HERON’s design, emphasizing lightweight implementations and enhanced interoperability.The HERON ontology was developed using Protégé version 5.6.1 (Stanford University, CA, United States), an open-source OWL editor. This version supports OWL 2, SHACL validation plugins, and SPARQL query integration, all of which were used in the current study. All relevant resources, including OWL ontology files, SHACL validations, and SPARQL queries, are openly available on GitHub https://github.com/ioannidou135/HERON-Ontology, (accessed on 28 April 2025) and Zenodo to ensure community reuse and extension (v1.0.2).

## 4. Discussion

### 4.1. HERON Ontology

The HERON ontology is a solution that addresses the longstanding complexities and usability challenges in healthcare robotics. By focusing on the streamlining of communication and collaboration modules, HERON achieves a balance between functional robustness and accessibility for diverse stakeholders. This refinement bridges the gap between theoretical frameworks and practical applications, making HERON a versatile tool for real-world deployment across various healthcare scenarios [6,17].

The updated communication module is designed to optimize how robots interpret and respond to natural human interactions. While no formal usability testing was conducted, the module’s architecture supports seamless integration into robotic workflows. For instance, in eldercare scenarios, HERON demonstrated its ability to facilitate dynamic, context-aware responses, enhancing the dialogue adaptability of robots to patients’ emotional and cognitive states. This capability is expected to improve user satisfaction, engagement, and adherence to care routines [8,22]. Similarly, the collaboration module addresses the inherent complexities of healthcare settings such as surgical environments. It enables real-time task allocation and enhances procedural precision by streamlining task execution, reducing redundancy, and improving operational efficiency [35].

### 4.2. Applications in Healthcare Robotics

The integration of HERON’s ontological framework into healthcare settings presents transformative opportunities across multiple care domains. Its four core components—communication, collaboration, safety, and robot classification—provide the foundation; however, its practical implementation must address specific clinical needs and workflows to realize its full potential.

#### 4.2.1. Communication Module Applications

HERON’s communication module enables structured information exchange between robotic systems and healthcare stakeholders, complementing existing ontology frameworks, such as HL7, and extending them to facilitate their adaption to robotics. This compliance with widespread communication protocols enables robots to interact directly with existing infrastructure, such as electronic health records (EHRs). For example, robots in nursing can be integrated with EHR systems to facilitate the recording of patient healthcare histories, ensuring continuity of care. This integration would allow robots to assist in tasks such as medication management and patient monitoring, thereby improving the efficiency of nursing services and enhancing communication between patients and healthcare professionals [49]. Moreover, previous studies have indicated that addressing barriers such as controllability and adaptability can enhance the usability of robotic systems, which can then leverage EHR data to tailor their interactions based on patient needs [50,51].

#### 4.2.2. Collaboration Module Applications

The collaboration module facilitates the orchestration of multi-agent interactions in healthcare settings. This module can equip robots with the ability to engage meaningfully with patients, tailoring responses to contextual cues such as emotional states and personal preferences. This fosters trust, companionship, and social interaction, addressing issues like social isolation and adherence to care routines. While these capabilities were validated through instantiation rather than real-world testing, the results suggest a significant potential to enhance patient outcomes in eldercare settings [32,36]. In surgical contexts, HERON’s collaborative framework demonstrates its capacity to improve coordination within surgical teams. Role-based control systems and real-time decision-making aids allow robots to assist surgeons in high-pressure environments, ensuring procedural precision and efficiency. Robots used in rehabilitation need to adjust their operation based on human force and intention during physical therapy sessions. Researchers have investigated EMG-based control for power-assist exoskeletons to enhance safe and intuitive interaction [12].

The advanced HRI methods, including impedance-based learning, provide HERON with potential enhancements to its collaboration and safety modules. The method described establishes force–position relationship models which allow robots to adjust to unfamiliar physical spaces without compromising human safety during interactions [52]. The integration of these methods into HERON would occur through the enhancement of its context modules with sensor-derived interaction parameters to enable adaptive behavior refinement under semantic control. The added functionality would allow HERON to connect symbolic reasoning with real-time physical compliance during collaborative manipulation and rehabilitation tasks.

#### 4.2.3. Safety Module Applications

HERON’s safety module addresses healthcare safety. For example, multi-directional excessive forces on the soft tissue level and musculoskeletal level are the most relevant hazards for rehabilitation robots [53]. Moreover, the overall technical complexity of robotic surgery systems creates unique cybersecurity risks and harms. For example, a breach of a surgical robot could lead to immediate and critical physical harm. Surgical data logs and patient videos are also points of attack, and breaches could compromise confidential preference data, surgeon-specific performance information, or patients’ personal health information [54]. Robotic ontologies can significantly help address security risks in healthcare settings by providing structured frameworks for understanding and managing various cybersecurity aspects as well as for fulfilling user-level, safety, or ethical requirements [29]. This includes model relationships between various elements in the cybersecurity domain, including medical devices, connectivity, vulnerabilities, and exploits [55].

#### 4.2.4. Robot Classification Module Applications

The classification module enables appropriate robot deployment through categorizing robots based on their clinical capabilities (e.g., medication delivery, patient transport, surgical assistance). By categorizing robots based on their characteristics, capabilities, and functionalities, healthcare providers can match specific robot types to appropriate tasks, ultimately improving operational efficiency and patient care. By providing a common vocabulary and structure, ontologies facilitate communication between different robotic systems and healthcare applications. For example, CORA serves as a foundational ontology that categorizes robots and their functionalities within healthcare settings. It defines key concepts such as robot parts, complex robots, and robotic systems, facilitating a common understanding across different robotic applications. For instance, CORA has been integrated into the ODIN project, which aims to create a semantic representation for robots used in daily hospital activities. This integration allows improved communication between robotic systems and healthcare applications, enhancing operational efficiency and safety in hospital environments [20,56].

### 4.3. Performance Metrics

The performance evaluation of HERON involved running SPARQL queries and SHACL constraints against agents that operated within simulated scenarios. The evaluation focused on ensuring semantic constraints applied correctly while enforcing them across different modules (e.g., collaboration, safety) rather than measuring timing or execution success rates. The system underwent evaluation through testing scenarios that included task delegation with role-based permissions and emergency escalation logic and ethical entry constraints for vulnerable patient contexts. Agents had to pass SHACL validation, which checked institutional policies (e.g., *hasAuthorityToOverride*, *requiresHumanEscort*) before performing any action. HERON successfully demonstrated semantically consistent and policy-compliant behavior across different healthcare workflows through its testing of complex inter-agent dependencies. Quantitative benchmarking is designated as future work.

The semantic robustness and domain fitness of HERON was assessed through qualitative evaluation criteria during scenario-based validation. Table 4 describes the core aspects examined, along with the mechanisms used for runtime reasoning and constraint checking.

The criteria demonstrate HERON’s capacity to support safe, policy-aware, and adaptive behavior in simulated healthcare workflows, thus validating its architectural soundness and practical readiness for real-time agent coordination. As summarized in Table 4, HERON was evaluated through scenario-based semantic validations, confirming its readiness for deployment under policy-compliant conditions.

The results show that HERON can enforce context-specific rules and semantic constraints in real-time, so that robotic agents can behave ethically and predictably even in uncertain healthcare environments. The validation process also further shows that HERON is practically ready as a policy compliant reasoning layer for complex multi-agent robotic deployments.

### 4.4. Comparison with Other Ontologies

A comparative analysis of HERON against other prominent ontology frameworks highlights its strengths in addressing the unique challenges of healthcare robotics. While each of these frameworks offers distinct advantages, HERON’s domain-specific modular design and focus on real-time interaction make it particularly suited to dynamic healthcare environments.

**SUMO (Suggested Upper Merged Ontology):** SUMO provides a general-purpose ontology framework with strong theoretical foundations, making it a valuable tool for AI and knowledge-based systems. However, its lack of domain specificity limits its effectiveness in addressing healthcare robotics’ nuanced demands. For instance, SUMO struggles with task execution and modularity in eldercare and surgical contexts, where HERON excels by facilitating real-time task allocation and collaboration [23,25,30].

**HL7 (Health Level Seven):** HL7 excels in healthcare data standardization and interoperability, particularly for managing electronic health records (EHRs). Its focus on enabling seamless data exchange between disparate systems makes it a critical tool for hospital data management. However, HL7 is less suited for dynamic applications involving robotic interactions and multi-agent systems. In contrast, HERON’s features, such as task management and context-aware communication, address these gaps, making it a more effective choice for high-stakes environments like surgical operations and emergency response [26,28,34].

**MIMO (Medical Informatics and Digital Health Multilingual Ontology):** MIMO offers unique strengths in supporting global healthcare collaboration through multilingual data representation, making it ideal for cross-border initiatives. However, MIMO lacks the adaptability required for dynamic and interactive robotics applications. HERON bridges this gap with its modular architecture, enabling real-time adaptability and coordination in complex healthcare scenarios while retaining linguistic and semantic precision [27,28,42].

Table 5 provides an overview of the comparisons between HERON and other ontology frameworks. Also, the table summarizes HERON’s comparative positioning with established frameworks, reflecting its operational and ethical strengths.

HERON’s novel modular design and healthcare-specific focus redefine seamless collaboration between robotic agents and human counterparts, offering capabilities unmatched by existing ontologies. These features address critical challenges, such as task allocation, error mitigation, and compliance with ethical standards. HERON also distinguishes itself by integrating ethical considerations into its core design, incorporating role-based access controls, data encryption, and GDPR compliance. This alignment with privacy and regulatory requirements ensures that HERON maintains stakeholder trust and confidence in healthcare applications [28,41].

While HERON demonstrates considerable advantages, challenges remain in scaling the framework for large, interconnected healthcare systems. The computational demands of HERON’s modular design, although mitigated through simplification, may still pose difficulties in resource-constrained environments. Additionally, ensuring seamless interoperability with diverse platforms and legacy systems requires ongoing research and refinement. Also, existing ontologies like SUMO and HL7 provide foundational frameworks, but they often lack easy accessibility for widespread adoption. In contrast, HERON’s public repository ensures transparency, collaboration, and ease of implementation. The inclusion of a DOI further supports its integration into research workflows by providing a citable and persistent resource. By combining modularity, real-time adaptability, and ethical alignment, HERON bridges the gap between theoretical ontology research and practical applications, emerging as a transformative framework for healthcare robotics.

The design and instantiation of HERON occurred with deployment in mind unlike numerous upper ontologies which stay abstract or application-agnostic. The modular structure allowed direct encoding of healthcare-specific roles and permissions and safety policies which could be tested against concrete tasks. HERON provided runtime feedback about agent behavior and task eligibility through its SPARQL reasoning and SHACL validation capabilities in realistic scenarios. The practical connection between conceptual design and semantic enforcement in HERON sets it apart from theoretical frameworks. The evaluated use cases of HERON show substantial progress toward ontology-driven autonomy in healthcare robotics although it does not function as a complete deployment stack.

#### Practical Readiness and Deployment Potential

The HERON system has not been deployed in a clinical robotic system yet, but its ontology design and semantic validation pipeline are technically aligned with integration in modular middleware and agent control layers. The current implementation supports real-time reasoning through SPARQL queries and constraint checking using SHACL, making it suitable for incorporation into behavior trees or decision-making layers of robot architectures. The clear modular boundaries of this system enable the deployment of communication, safety, or collaboration reasoning subsystems as separate entities based on robotic role or institutional context. The characteristics of HERON indicate its potential to function as an intermediary layer which connects abstract knowledge modeling to operational task execution thus bridging theoretical ontologies with usable healthcare robotics frameworks.

The computational overhead from modular reasoning presents deployment difficulties in low-resource settings such as small clinics. Future versions of HERON should investigate lightweight constraint abstraction and precompiled validation modules and ontology compression methods to achieve real-time operation with limited hardware resources.

### 4.5. Future Research Directions

HERON’s potential offers numerous avenues for future research and development. One critical priority is addressing scalability challenges. While HERON has shown success in individual applications, further research is needed to adapt its framework for large, interconnected healthcare systems. This includes developing advanced algorithms for real-time multi-agent coordination, resource allocation, and conflict resolution in dynamic environments [33,57]. Future versions of the ontology may need lightweight ontology compression or precompiled reasoning components to make them more suitable for low-resource hospital settings. The feasibility of lightweight HERON implementations for clinics with limited IT infrastructure should also be explored, as computational constraints may prevent real-time deployment. In such contexts, resource allocation and conflict resolution remain critical factors for maintaining autonomy under strict performance budgets.

The integration of IoT-enabled healthcare robotics presents another promising opportunity. IoT devices, such as wearable sensors and smart hospital infrastructure, generate real-time data streams that HERON could leverage to enhance decision making and care delivery. For example, eldercare robots could use IoT data to monitor patients’ vital signs continuously, enabling proactive interventions and personalized care plans [36,42].

AI-driven diagnostics and predictive analytics also represent an exciting frontier for HERON’s application. By incorporating advanced AI techniques, HERON could improve its ability to process complex datasets, allowing robots to provide more accurate and timely support in critical healthcare scenarios. Future research must address the ethical implications of these advancements, particularly in maintaining transparency, ensuring accountability, and aligning autonomous decisions with societal values [45,46,48].

HERON’s ethical framework will require ongoing evolution to accommodate emerging technologies such as quantum computing and advanced AI. These innovations introduce new privacy and security challenges, necessitating continuous updates to HERON’s design. Mechanisms such as explainable AI, enhanced transparency, and adaptive role-based access controls will be critical for maintaining stakeholder trust and fostering acceptance in increasingly complex healthcare ecosystems [48,58].

#### Towards Integrated Semantic Middleware

The current framework of HERON enables future integration with robotic middleware systems and healthcare data infrastructure beyond its current semantic modeling capabilities. The system can connect to real-time robotic systems through ROS 2 platforms to convert validated semantic plans into actionable behaviors. The research aims to integrate healthcare interoperability standards which include HL7 and FHIR. The integration of HERON classes with structured healthcare data through semantic mappings would enable policy-aware robot behavior that draws from clinical states and alerts and contextual triggers. SHACL functions mainly for constraint validation yet its integration with temporal reasoning mechanisms and SPARQL-based context prediction allows proactive decision making in dynamic care environments. The proposed directions transform HERON from theoretical concepts into a practical semantic framework which supports accountable autonomous healthcare robotics.

## 5. Conclusions

This research introduces HERON, an ontology for healthcare robots that contributes to their seamless integration into healthcare systems. As an upper ontology, HERON provides a framework capable of addressing the diverse and dynamic challenges of healthcare robotics. Its modularity allows it to support specific applications, while its ethical alignment and regulatory compliance ensure its applicability in sensitive and high-stakes environments.

HERON’s ability to balance complexity with usability can enhance both patient outcomes and operational efficiency. Continued interdisciplinary collaboration and innovation will be critical to refining HERON’s capabilities and ensuring its sustained relevance in the rapidly evolving landscape of healthcare technology.

The current framework of HERON enables future integration with adaptive control strategies used in physical HRI settings that employ impedance-based methods. The modular design of HERON enables its application in mental health robotics as well as remote diagnostic agents and AI-enhanced clinical support tools. Future studies will investigate how HERON can integrate with large language model (LLM)-driven robotic interfaces to create hybrid symbolic–neural reasoning layers in delicate healthcare settings. The research directions will develop HERON into both a semantic backbone and an adaptive scalable enabler of next-generation robotic autonomy.

The HERON ontology can bridge the gap between theoretical advancements and practical implementation. By aligning scalability, ethical considerations, and real-world adaptability, HERON lays the foundation for the next generation of intelligent and reliable robotic systems in healthcare. The current privacy policies of the ontology follow GDPR guidelines, but its modular structure allows easy adaptation to different regulatory frameworks, which enables global applicability across various legal systems. The ENDORSE project represents only one application of HERON because this framework shows potential for use in emergency triage coordination as well as elderly telecare systems and remote diagnostics in underserved regions. The scenarios need decentralized semantically robust decision-making frameworks which HERON can support through its modular instantiations and validation rules. Mobile triage units and AI-assisted mental health support agents will use HERON’s ethical reasoning layer to guide their operations in future applications. The validation of HERON ontology through semantic constraint enforcement and role-context reasoning across instantiated scenarios demonstrates its practical readiness for integration into robotic middleware and safety-critical healthcare workflows.

## Figures and Tables

**Table 1:** Examples of healthcare robotics applications.

Application Domain	Example Systems	Key Features	Impact	Sources
Eldercare	Paro Robot, Care-O-Bot, Remote-Controlled Service Robot	Social interaction, health monitoring, assistance	Reduces loneliness, enhances quality of life	[1,2,5,7]
Surgery	Da Vinci Surgical System	Minimally invasive procedures, high precision	Shortens recovery time, reduces complications	[3,4,8]
Rehabilitation	Lokomat, ReWalk, EMG-Controlled Exoskeleton	Motor function recovery, gait training	Improves patient mobility and independence	[9,10,11,12]
Patient Monitoring	TUG Robots, Robear	Vital signs tracking, mobility assistance	Enhances efficiency in patient care	[13,14,15]
Hospital Logistics	Aethon TUG, Moxi	Transport of supplies, medications, and waste	Reduces staff workload, increases operational efficiency	[16,17]

**Table 2:** Comparison of ontology frameworks.

Framework	Focus Area	Strengths	Weaknesses	Sources
HERON	Healthcare robotics	Modular design; supports communication, collaboration, and safety	Computational complexity; resource-intensive	[23,24,29]
SUMO	General ontology integration	Broad applicability; foundational framework	Limited domain specificity	[25,30]
HL7	Healthcare data interoperability	Standardization of healthcare information	Narrow scope; lacks adaptability for robotics	[26,31]
OBO	Biomedical data standardization	Strong in data accuracy and ontology alignment	Ineffective in multi-agent task management	[27,28,31]

**Table 3:** Metrics for evaluating the HERON ontology.

Metric	Description	Evaluation Methods	Sources
Instantiation Validity	Accuracy and consistency of HERON’s representations of safety-critical and operational factors.	Alignment with meta-model specifications and ENDORSE project requirements.	[8,10]
Alignment with Standards	Adherence to GDPR, role-based access control, and encryption requirements for sensitive data.	Validation through comparison with regulatory standards and stakeholder recommendations.	[10,33,47]
Scalability	Ability to adapt HERON to various robotic systems and healthcare workflows in different contexts.	Analysis of instantiation outcomes in eldercare scenarios and hypothetical surgical environments	[8,16,28]

**Table 4:** Key evaluation criteria used for HERON validation.

Evaluation Area	Description	Validation Mechanism
Task Eligibility	Evaluation of agent role, patient state, and task-specific context during instantiation	SPARQL queries
Safety and Policy Compliance	Verification of permission rules, risk conditions, and override logic	SHACL shapes
Collaboration Flow	Delegation between agents under institutional constraints	SPARQL + SHACL coordination
Adaptability	Scenario-dependent reasoning for task adjustment and escalation conditions	Modular ontology instantiation

**Table 5:** HERON vs. other ontology frameworks.

Framework	Strengths	Weaknesses	Application Areas	Sources
HERON	Enhanced modularity; robust communication and collaboration; aligned with healthcare robotics needs	Scalability challenges in large-scale systems; computational resource demands	Eldercare, surgical robotics, real-time interaction scenarios	[23,26,28,42,48]
SUMO	Broad general-purpose ontology; strong theoretical foundations	Limited domain specificity; less suited for dynamic healthcare scenarios	General AI, foundational ontology research	[25,30]
HL7	Healthcare data standardization; interoperability between systems	Focus on static data interoperability; lacks robotic interaction capabilities	Hospital data management, electronic health records	[26,31,34,48]
MIMO	Multilingual support for global healthcare data; inclusivity	Insufficient adaptability for dynamic tasks in healthcare robotics	Cross-border healthcare data initiatives, global collaboration	[27,28,42]

## Data Availability

The HERON ontology has been made publicly accessible on Zenodo (https://doi.org/10.5281/zenodo.15116119, last accessed 31 March 2025) under an open-access license.
